# Recurrent *C. difficile* in a Patient with IgG Deficiency

**DOI:** 10.1155/2015/356293

**Published:** 2015-04-05

**Authors:** Asad Jehangir, Kyle Bennett, Shoaib Bilal Fareedy, Andrew Rettew, Bilal Shaikh, Anam Qureshi, Qasim Jehangir, Richard Alweis

**Affiliations:** ^1^Department of Internal Medicine, Reading Health System, 6th Avenue and Spruce Street, West Reading, PA 19610, USA; ^2^King Edward Medical University, Mayo Hospital Road, Nelagumbad, Anarkali, Lahore 54000, Pakistan; ^3^Rawalpindi Medical College, Tipu Road, Rawalpindi 46000, Pakistan; ^4^Sidney Kimmel Medical College, Thomas Jefferson University, 1025 Walnut Street, Philadelphia, PA 19107, USA

## Abstract

IgG deficiency can predispose to recurrent pyogenic infections. The association of IgG deficiency with *Clostridium difficile* infection has been infrequently reported in the literature. We present a case of a middle-age woman with multiple hospitalizations for recurrent *C. difficile* in a short span of time which prompted consideration of a possible fecal transplant. On evaluation, she was found to have low total IgG, with subclass analysis revealing low IgG1 and IgG3. She was started on monthly infusions of immunoglobulins and one year after her last episode of *C. difficile* she has not had any recurrence. The role of immunoglobulin infusion in the treatment of recurrent *C. difficile* is controversial, with some studies revealing no clear evidence of benefit. Our case report suggests that the patients who have underlying IgG deficiency may benefit from immunoglobulin, as this can significantly reduce the incidence of recurrent infections and hence save the healthcare costs.

## 1. Introduction

Low levels of immunoglobulin G (IgG) or one of its subclasses can be detected on laboratory testing in up to 20% of the population but predispose only a small subset of these patients to recurrent pyogenic infections like* Streptococcus pneumonia* [1, 2]. The association of IgG deficiency with* Clostridium difficile* has been rarely reported in the literature. We present a case of a middle-aged female with 3 episodes of* C. difficile* colitis within a 4-month period who was found to have IgG1 and IgG3 deficiency on evaluation and had no recurrences after the initiation of immunoglobulin infusions (IVIG).

## 2. Case Report

A 50-year-old female with past medical history of asthma, hyperlipidemia, and anxiety was admitted to the hospital with complaints of profuse diarrhea with up to 30 loose bowel movements a day. She also complained of loss of appetite and lower abdominal discomfort that improved with defecation. She denied any recent sick contacts or exposure to unusual food. Her home medications included montelukast 10 mg nightly, albuterol inhaler when needed, fenofibrate 135 mg daily, and diazepam 10 mg four times daily. On examination, she was afebrile and normotensive but tachycardic with pulse of 100. Abdominal examination revealed mild epigastric tenderness. Laboratory tests revealed leukocytosis with white cell count of 15,600 per mcL and a normal comprehensive metabolic panel and lipase. A CT abdomen and pelvis with and without contrast was unremarkable and showed normal colon, small intestine, liver, and gall bladder. The stool studies including fecal leukocytes,* Campylobacter*,* Salmonella*,* Shigella*,* Cryptosporidium*,* Giardia*, ova, and parasites were normal. However, stool* Clostridium difficile* toxin test was positive. The patient failed to improve from the initial treatment with metronidazole and was switched to oral vancomycin, to which she responded well with resolution of diarrhea. She was discharged home on a probiotic (*Saccharomyces boulardii* 250 mg twice daily). A few weeks later she was hospitalized again with a recurrence of* C. difficile* diarrhea and was treated with a 2nd course of vancomycin with symptomatic improvement. About 2 months later she had her 3rd episode of* C. difficile* diarrhea for which was prescribed vancomycin with a prolonged taper. The patient continued to have abdominal discomfort and diarrhea even on vancomycin and because of multiple recurrences of* C. difficile*, she was being considered for a fecal transplant. However, the repeat* C. difficile* toxin was negative; hence the fecal transplant was not performed.

The patient had an extensive evaluation to determine the cause of persistent diarrhea with urine 5-hydroxyindoleacetic acid and chromogranin A, esophagogastroduodenoscopy, endoscopic ultrasound (to visualize pancreas, gall bladder, and liver) CT enterography, and a colonoscopy with random biopsies, all of which were unremarkable. Her symptoms were felt to be related to postinfectious diarrhea predominant irritable bowel syndrome for which she started on alosetron, which resulted in resolution of diarrhea. In the meantime, she was also evaluated for a possible immunoglobulin deficiency which revealed a normal IgA of 188 mg/dL (reference range 61 to 356 mg/dL), IgM of 92 mg/dL (reference range 37 to 286 mg/dL), and IgE of 39 IU/mL (reference range 1 to 165 IU/mL). However, IgG was found to be low at 661 mg/dL (reference range 767 to 1590 mg/dL). IgG subclasses showed low IgG1 of 229 mg/dL (reference range 341 to 894 mg/dL) and low IgG3 of 13.8 mg/dL (reference range 18.4 to 106 mg/dL), whereas IgG2 and IgG4 were normal. IgG deficiency was later confirmed with a repeat laboratory test and she was started on monthly immunoglobulin infusions for IgG deficiency. One year after her last* C. difficile* infection, she continues to receive monthly immunoglobulin infusions and has not developed any recurrence since then.

## 3. Discussion

IgG is the most prevalent immunoglobulin (IG) in the human body and is comprised of 4 subclasses: IgG1, IgG2, IgG3, and IgG4. The normal levels of IgG vary widely and up to 1/5th of the population may have low levels of one or more subclasses of IgG, which is defined as more than 2 standard deviations below normal [1]. However, there should also be concurrent evidence of recurrent infections or impaired response to protein and/or polysaccharide vaccinations in such cases to label them as IgG deficient. In our patient, low levels of IgG confirmed on repeat testing along with 3 episodes of* C. difficile colitis* in a short interval of time helped us establish a diagnosis of IgG deficiency.

IgG1 comprises approximately 2/3rd of the total serum IgG; hence, its deficiency generally corelates with low total serum IgG. IgG3 constitutes 4–8% of the total serum IgG and deficiency in this subclass is commonly seen in concern with IgG1 [3]. In a study of 503 patients with subclass deficiencies, IgG3 subclass deficiency was the most common and had a female preponderance [4]. IgG1 and IgG3 deficiencies predispose to recurrent infections, in particular sinopulmonary infections [2, 3]. This increased susceptibility may be explained by the underlying T and B cell functional deficits found in majority of these patients [5].

Generally, IgA antibodies are presumed to contribute to most of the immunity against gastrointestinal infections [6]. Hence, the association of IgG antibodies with recurrent* C. difficile* infection has been infrequently reported in the literature. There have been very few case reports of IgG deficiency in patients with recurrent* C. difficile colitis* that improved with immunoglobulin replacement [6]. However, in a study from 2007, similar levels of total IgG and IgG subclasses were demonstrated in patients with isolated and recurrent* C. difficile* infection, even though low levels of IgG2 and IgG3 antibodies to* C. difficile* antitoxin A were found in patients with recurrent* C. difficile* [7]. Our case report suggests that patients with recurrent* C. difficile* infection may have previously undiagnosed IgG subclass deficiency and early recognition of this with appropriate treatment may prevent further recurrences.

In patients with IgG deficiency with evidence of recurrent infections, intravenous or subcutaneous immunoglobulin replacement significantly improved the quality of life, reduced the incidence of infections, and normalized the levels of IgG antibodies [8]. The decreased incidence of infection likely results from the passive transfer of antibodies against the invading microbes [3]. The role of immunoglobulins in the treatment of chronic relapsing* C. difficile* colitis is controversial. There are some case reports that suggest that such patients may benefit from immunoglobulins [9, 10]. However, a retrospective study failed to demonstrate any clear improvement from immunoglobulins in patients with* C. difficile* colitis [11]. Our case report proposes that recurrent* C. difficile* from underlying IgG deficiency could benefit from regular immunoglobulin infusions.

## 4. Conclusion

Considering possible IgG deficiency in patients with recurrent episodes of* Clostridium difficile* infection, timely diagnosis and initiation of immunoglobulins can markedly decrease the chances of recurrence of* C. difficile* colitis. This can potentially lessen the exposure to antibiotics, reduce the chances of complications and hospitalizations, and save healthcare costs.
